# Fluorescence *In Vivo* Hybridization (FIVH) for Detection of *Helicobacter pylori* Infection in a C57BL/6 Mouse Model

**DOI:** 10.1371/journal.pone.0148353

**Published:** 2016-02-05

**Authors:** Sílvia Fontenete, Marina Leite, Davie Cappoen, Rita Santos, Chris Van Ginneken, Céu Figueiredo, Jesper Wengel, Paul Cos, Nuno Filipe Azevedo

**Affiliations:** 1 LEPABE, Laboratory for Process Engineering, Environment, Biotechnology and Energy, Faculty of Engineering, University of Porto, Porto, Portugal; 2 i3S, Instituto de Investigação e Inovação em Saúde, Universidade do Porto, Porto, Portugal; 3 IPATIMUP, Institute of Molecular Pathology and Immunology of the University of Porto, Porto, Portugal; 4 Nucleic Acid Center, Department of Physics, Chemistry and Pharmacy, University of Southern Denmark, Odense M, Denmark; 5 ICBAS, Institute of Biomedical Sciences Abel Salazar, University of Porto, Porto, Portugal; 6 Laboratory of Microbiology, Parasitology and Hygiene (LMPH), Faculty of Pharmaceutical, Biomedical and Veterinary Sciences, University of Antwerp, Antwerp, Belgium; 7 Laboratory of General Biochemistry and Physical Pharmacy, Ghent University, Gent, Belgium; 8 Laboratory of Applied Veterinary Morphology, Faculty of Pharmaceutical, Biomedical and Veterinary Sciences, University of Antwerp, Antwerp, Belgium; 9 FMUP, Faculty of Medicine of the University of Porto, University, Porto, Portugal; Cornell University, UNITED STATES

## Abstract

**Introduction:**

In this study, we applied fluorescence *in vivo* hybridization (FIVH) using locked nucleic acid (LNA) probes targeting the bacterial rRNA gene for in vivo detection of H. pylori infecting the C57BL/6 mouse model. A previously designed Cy3_HP_LNA/2OMe_PS probe, complementary to a sequence of the *H*. *pylori* 16S rRNA gene, was used. First, the potential cytotoxicity and genotoxicity of the probe was assessed by commercial assays. Further, the performance of the probe for detecting *H*. *pylori* at different pH conditions was tested *in vitro*, using fluorescence *in situ* hybridization (FISH). Finally, the efficiency of FIVH to detect *H*. *pylori* SS1 strain in C57BL/6 infected mice was evaluated *ex vivo* in mucus samples, in cryosections and paraffin-embedded sections by epifluorescence and confocal microscopy.

**Results:**

*H*. *pylori SS1* strain infecting C57BL/6 mice was successfully detected by the Cy3_HP_LNA/2OMe_PS probe in the mucus, attached to gastric epithelial cells and colonizing the gastric pits. The specificity of the probe for *H*. *pylori* was confirmed by microscopy.

**Conclusions:**

In the future this methodology can be used in combination with a confocal laser endomicroscope for *in vivo* diagnosis of *H*. *pylori* infection using fluorescent LNA probes, which would be helpful to obtain an immediate diagnosis. Our results proved for the first time that FIVH method is applicable inside the body of a higher-order animal.

## Introduction

*Helicobacter pylori* (*H*. *pylori*) colonizes the human gastric epithelium, representing the most common infection worldwide [1]. This infection increases the risk for peptic ulcer disease, distal gastric adenocarcinoma and mucosa-associated lymphoid tissue lymphoma [1,2]. Due to its important role in gastric cancer development, *H*. *pylori* was recognised as a carcinogen (class 1) by the World Health Organization [3]. Therefore, a rapid, accurate and early diagnosis of *H*. *pylori* infection is crucial not only for individual patient management but also to identify individuals at high risk of developing gastric cancer. Currently, a number of diagnostic methods are available to detect *H*. *pylori* [4]. Upper endoscopy allows the collection of gastric biopsy specimens used to identify *H*. *pylori* by histology, culture, rapid urease tests, and PCR-based methods [3]. Therefore, and with the exception of rapid urease tests, the diagnostic result is not immediately obtained after endoscopy, requiring time and an experienced laboratory.

Confocal laser endomicroscopy (CLE) allows *in vivo* visualization and analysis of epithelial mucosa using 1000x magnification [5]. Some studies have suggested that CLE images of colonic mucosa have the potential to substitute conventional histological diagnostics [5,6,7]. However, only unspecific stains, such as fluorescein sodium and acriflavine have been used for *in vivo* histology of the mucosal layer [8,9,10]. For this reason, only indirect evidence of the presence of *H*. *pylori* infection can be observed [11]. Consequently, a precise and specific identification of this bacterium is not yet possible using this method.

Fluorescence *in vivo* hybridization (FIVH) can be applied in detecting DNA or RNA sequences in living eukaryotic cells [12]. Nevertheless, to the best of our knowledge this method was never employed to detect microorganisms directly in the human body, possibly due to the peptidoglycan cell wall of the microorganisms that hinders the entry of the probes into the cell. Nucleic acid mimics such as peptide nucleic acid (PNA), locked nucleic acid (LNA) and 2’-O-methyl-RNA (2OMe) have been studied as diagnostic probes of infectious diseases and are capable of replacing conventional DNA or RNA probes [13]. The key advantages of these mimics are the higher stability *in vivo* [14,15,16] and the more favorable diffusion and hybridization properties than the corresponding unmodified DNA or RNA probes [17,18]. However, the unsolved question of how these types of probes can be used to detect clinically relevant bacteria remains. The different approaches that have been undertaken to develop *in vivo* diagnostic methods mostly rely on the use of non-specific stains or label oligonucleotides with radioactive isotopes such as halogens [19,20,21].

We recently reported the development of a LNA probe (HP_LNA/2OMe_PS) that specifically detects *H*. *pylori* in biopsies of infected patients, and in experimental conditions of extreme acid pH and at 37°C [22,23]. Still, the performance of this probe for *H*. *pylori in vivo* detection remained unknown.

Here, we provide the first FIVH protocol applied to an experimental mouse model of *H*. *pylori* infection, that successfully enables the detection of both free-swimming bacteria in the protective mucus layer that overlays the stomach surface and bacteria colonizing gastric epithelial cells.

## Materials and Methods

### Oligonucleotide synthesis

The sequence of the probe was selected and synthesized based on the parameters described in our previous studies [22,23] (Table 1).

**Table 1:** Designation and sequence of probe containing locked nucleic acid (LNA; with L superscript) and 2’-O-methyl-RNA (2’-OMe; in boldface) nucleotide monomers. Cy3_HP_LNA/2OMe_PS is a phosphorothioate oligomer (PS backbones), Cy3 (Cyanine).

Designation	Sequence (10-mer)
Cy3 HP_ LNA/2OMe _PS	5'- Cy3 G^L^**AC**T^L^**AA**G^L^**CC**C^L^-3’

### Cell proliferation assay

The human gastric epithelial cell line AGS (ATCC^**®**^ CRL-1739) was cultured in RPMI medium 1640 Glutamax I (Gibco, Invitrogen, Grand Island, NY, USA) supplemented with 10% (v/v) fetal bovine serum (FBS, HyClone, Thermo Fisher Scientific, Inc, UK) and 1% (v/v) Penicillin/Streptomycin (Gibco) at 37°C in a humidified 5% CO_2_ atmosphere. Cells were seeded at 1.6 x 10^5^ cells/well in a 96-well plate. After overnight, media were changed and cells were incubated with predetermined concentrations (0.4 μM to 2 μM) of Cy3_HP_ LNA/2OMe _PS probe diluted in vehicle (0.5 M urea and 900 mM NaCl) or only with the vehicle, for 24 h. Cell viability was assessed by CellTilter 96^®^ Aqueous One Solution Cell Proliferation Assay (Promega Corporation, Madison, WI), according to the manufacturer’s instructions. Untreated cells served as a negative control. The experiments were performed in triplicate.

### VITOTOX ®Assay

To analyze if the Cy3_HyP_LNA/2’OMe probe can induce genotoxicity, the VITOTOX^®^ model from Gentaur (Aachen, Germany) was used. Briefly, this assay makes use of two recombinant *Salmonella typhymurium* reporter strains, the TA104 recN2-4 (Genox strain) and TA104 pr1 (Cytox strain). The Cytox strain expresses bacterial luciferase from *Vibrio fischeri*, encoded episomal. A reduction in the signal/noise of the luminescent signal indicates cytotoxicity and an increase indicates interferences of the compound with the luminescent signal itself. The Genox strain carries an integrated lux operon from *Vibrio fischeri* under transcriptional control of the recN promoter. When a compound is genotoxic, transcription of the DNA repair mechanism will lead to an increase of the luminescent signal. Addition of rat liver S9 fraction is used to mimic the mammalian metabolic conditions so that the mutagenic potential of metabolites formed by a parent compound in the hepatic system can be assessed [24,25,26]. The luminescence is measured for 4 h with a 5 min interval period in a plate-reading GloMax® Discover System GM3000 luminometer (Promega, Madison, USA) and generally, when the signal to noise ratio in the cytox strain model is reduced below 0.8, the compound is regarded as cytotoxic for *S*. *typhymurium* and the genotoxicity cannot be studied. When the signal to noise ratio in the genox strain model is above 1.5, the DNA repair mechanism is activated by the cell as an early response to genotoxicity by the compound. The concentrations of Cy3 HP_LNA/2OMe_PS probe tested in this assay were of 0.04, 0.08, 0.2, 0.4, 1 and 2 μM. The compound 4-nitroquinoline-oxide (4-NQO) (a direct acting base-altering mutagen) and benzo[α]pyrene (BaP) (indirect mutagen which requires metabolic activation by S9 mix), were used as positive controls. The experiments were performed in triplicate.

### Bacteria and growth conditions

The mouse-adapted *Helicobacter pylori* Sydney strain 1 (SS1), originally described by Lee *et al*., [27], was kindly provided by Sara Lindén (Gothenburg University, Sweden). *H*. *pylori* SS1 was routinely cultured for 48 h on Tryptic Soy Agar (TSA) plates (Lab M Limited, Lancashire, UK) medium, supplemented with 5% sheep blood (Oxoid, Cambridge, UK) at 37°C under micro-aerophilic conditions (5% O_2_, 10% CO_2_, 85% N_2_), generated by Whitley H35 Hypoxystation (Don Whitley, West Yorkshire, UK). For liquid cultures, bacteria were resuspended in Tryptic Soy Broth (TSB) (Lab M Limited, Lancashire, UK) containing 10% of fetal calf serum (FCS) (Invitrogen, Ghent, Belgium) and grown overnight at 37°C, with shaking under microaerophilic conditions.

### Fluorescence in situ hybridization on slides

To determine if the probe Cy3-labeled HP_LNA/2OMe_PS detect the *H*. *pylori* SS1 strain, a theoretical evaluation was performed against the 16 S rRNA from this bacterial strain, using BLAST software (http://blast.ncbi.nlm.nih.gov/Blast.cgi). Then, the hybridization of the Cy3 HP_LNA/2OMe_PS against *H*. *pylori SS1* was studied using LNA-FISH protocol on glass slides as previously described by Fontenete *et al*., (2015), with few modifications. Briefly, 20 μL of *H*. *pylori SS1* liquid cultures were spread onto glass slides and the smears were allowed to air dry. The hybridization was performed using 20 μL of hybridization buffer with 0.2 μM of the probe, which covered each smear individually. Three different hybridization buffers were tested at different pH (2, 4, and 7), containing 0.5 M urea (BHD Prolabo, Haasrode, Belgium), 900 mM NaCl (Panreac, Illinois, USA) and different buffer solutions to keep the desired pH (pH 2: KCl-HCl, pH 4: phosphate-citrate, pH 7: Tris-HCl buffer). Smears were covered with coverslips and incubated for 30 min at 37°C. Slides were subsequently washed in a gastric simulated juice that contains pepsin for 15 min at 37°C and then, the slides were allowed to air dry. All experiments were performed in triplicate and for each experiment a negative control (without a probe) was included. For image acquisition a Carl Zeiss inverted Axiovert fluorescence microscope (Carl Zeiss, Jena, Germany) was used. Cy3-labeling was excited by using a 565 nm laser; the exposure time was fixed for all preparations.

### Animals

Female specific-pathogen-free (SPF) from the inbred C57BL/6 (C57BL/6JRj strain) mice (*n* = 24) were purchased from Janvier LABS (Le Genest-St-Isle, France). Animals were housed in 332 × 150 × 130 cm (3 mice) or 382 × 220 × 150 cm (6 mice) autoclaved Micro-Isolater clear plastic cages, with a ventilation rate of 10 to 15 air changes per hour (ACH), at 20°C, 50% humidity and under a light/dark cycle of 12/12 h, with free access to standard rodent food pellets (Carfil Quality, Turnhout, Belgium) and water. Cages were lined with B 8/20 chips for bedding material, and with cardboard tubes and shredded paper as nesting material for environmental enrichment. Animals were handled by trained and experienced personnel for routine maintenance and experimental procedures to reduce stress. To minimize variation in the gut microflora, all animals within an experimental cohort were bred in the same room and housed on the same rack in a specific pathogen-free barrier facility. All animal experimentation was performed according to institutional guidelines and with approval of the local institute review board, in accordance to the European Directive 2010/63/EU regulations.

### Infection of mice with *H*. *pylori* SS1

**Fig 1:**
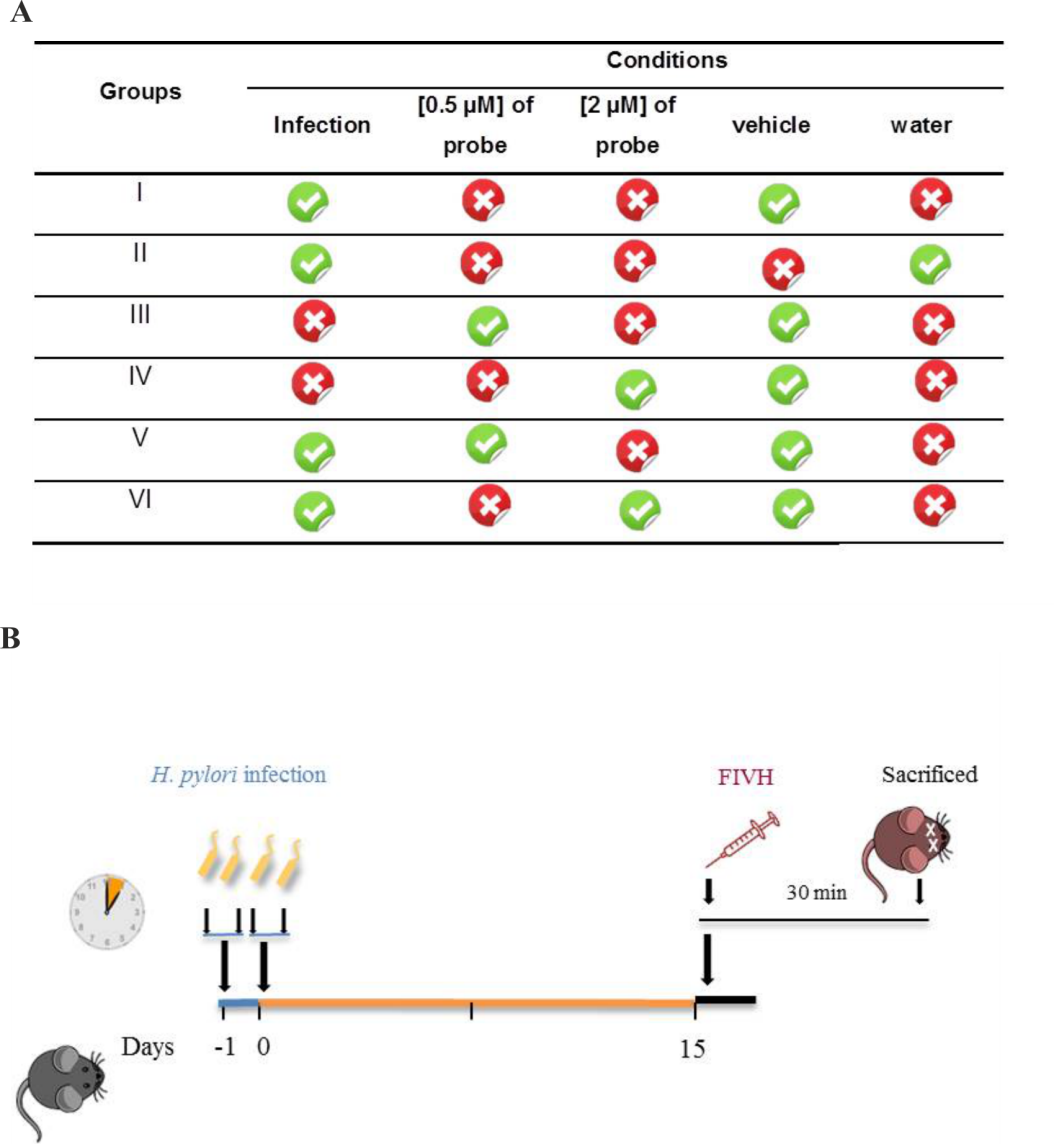
FIVH scheme used for detecting *H*. *pylori* in C57BL/6 mice. A. Four control groups with *n* = 3 (group I to IV) were used to evaluate the background of the vehicle (group I) and tissue (group II) and the specificity of the Cy3_HyP_LNA/2’OMe probe (groups III and IV). Two tests groups (*n* = 6, each group) were used with different probe concentrations, 0.5 μM (group V) and 2 μM (group VII). B. Animal study protocol depicting *H*. *pylori* inoculation, time of infection in C57BL/6 mice, and FIVH after 15 days post-infection.

Female SPF C57BL/6 mice were randomly allocated to 6 groups of 3 to 6 animals (Fig 1A). Mice were inoculated intragastrically with two doses of 0.1 mL TSB containing 1x10^9^ *H*. *pylori* CFU/mL by oral gavage (with polyethylene catheters attached to 1 mL disposable syringes, Biotrol, Paris, France). The administration was performed at two time points, with an interval of 1 hour, in two consecutive days. In the control groups, mice were given TSB alone. Mice were fasted from 4 h before infection until 4 h after oral gavage. Mice were sacrificed by cervical dislocation, 2 weeks post-infection for collection of stomach and detection of *H*. *pylori* by FIVH.

### FlVH procedure and assessment in mice

FIVH procedure was performed 15 days post-infection (Fig 1A). Cy3-labeled HyP_LNA/2’OMe probe, diluted in an adjuvant buffer contained 0.5 M urea and 900 mM NaCl), was given by oral gavage at 0.5 μM (group I and group V) or 2 μM concentration (group II and group VI) (Fig 1B). After 30 min, animals were sacrificed by cervical dislocation (Fig 1A). The stomach of each animal was removed in aseptic conditions and opened along the greater curvature.

The stomach contents were removed and samples of the mucus were recovered in coverslips. After that, the glandular stomachs were washed in physiological buffered saline and divided into tissue fragments representing cardia, body and antrum. Half of the stomach was used for *H*. *pylori* culture. The remaining half of the glandular stomach was equally divided in two parts. One part was rinsed in PBS with 0.01%NaN_3_ and immediately frozen in liquid nitrogen in Optimal Cutting Temperature compound (OCT; Sakura FInetek, USA) for histopathological examination and analysis of fluorescence. Tissue cryosections (10 μm) were prepared. The other part of the stomach was fixed in 4% paraformaldehyde (PAF, Sigma-Aldrich), for 1 hour, at room temperature. Afterwards, the tissue was rinsed 3 times for PBS and stored in PBS with 0.01% NaN_3_ at 4°C, before being processed and embedded in paraffin. Tissue sections with 3 μm were obtained.

The detection of FIVH signal was performed *ex vivo* in mucus samples, paraffin-embedded sections and cryosections. Mucus and cryosections were evaluated using a Carl Zeiss inverted Axiovert fluorescence microscope. Paraffin-embedded sections were evaluated using a Nikon Eclipse Ti-E inverted microscope attached to a microlens-enhanced dual spinning disk confocal system (UltraVIEW VoX; PerkinElmer, Seer Green, UK) equipped with 405, 488 and 561 nm diode lasers for excitation of blue, green and red fluorophores, respectively. Images were acquired and processed using Volocity image analysis software (Improvision, PerkinElmer, Waltham, USA).

### Retention of Cy3 HP_LNA/2OMe_PS probe in the mouse stomach

To evaluate the retention of the probe in the gastric mucosa, an extra experiment was performed using a group of mice with a period of 5 days post-infection. The infection procedure was performed as described in section 8. Each mouse in the group was administered 2 μM of Cy3_HyP_LNA/2’OMe probe through oral gavage. Mice were killed after 24 h and the stomach were processed and analyzed as previously described.

### Evaluation of *H*. *pylori* colonization in infected mice

The presence of *H*. *pylori* infection was determined by viable bacterial counts (CFU, colony forming units) and histopathological evaluation. To quantify bacteria on stomachs samples, as a measure of the colonization level, tissues fragments were mechanically homogenized in 1 mL TSB (TissueRuptor, QIAgen). The homogenates were serially diluted and then plated in duplicate onto TSA plates, supplemented with 5% sheep blood, vancomycin (10 μg/mL), trimethroprim (5 μg/mL), amphotericin (5 μg/mL) and cefsulodin (10 μg/mL) (all purchased from Sigma-Aldrich) and incubated at 37°C under microaerophilic conditions. After 5 days of incubation, *H*. *pylori* colonies were identified and enumerated as CFU per gram of stomach.

### Statistical analysis

Statistical significance was determined by One-way analysis of variance (ANOVA) using GraphPad PRISM 5 software (GraphPad Software, San Diego, USA). Results were expressed as mean ± SD. Differences were considered to be statistically significant when *p* < 0.05.

## Results

### HyP_LNA/2’OMe probe in vitro toxicity study

**Fig 2:**
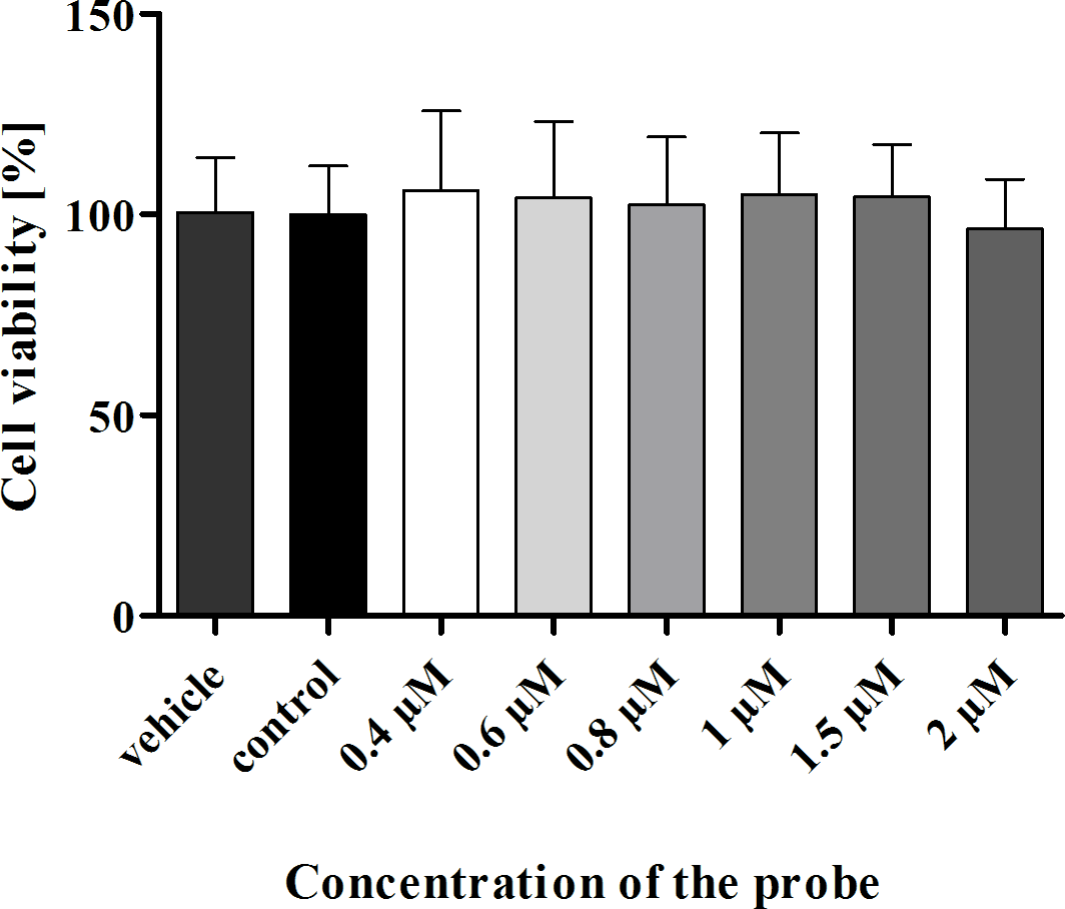
Effect of the Cy3_HP_LNA/2OMe_PS probe on viability of AGS gastric epithelial cells using the MTS assay. AGS cells were treated with a range of concentrations of the Cy3_HP_LNA/2OMe_PS probe for 24 h (0.4 μM to 2 μM). Results are expressed as the mean ± SEM of three independent experiments, performed in triplicate; No statistical significant differences were found regarding probe-treated *vs* untreated control cells (*p>0*.*05* by ANOVA).

To address if the Cy3-labelled HP_LNA/2OMe_PS probe affects gastric cell viability, we incubated a gastric epithelial cell line, AGS, with different concentrations of the probe and evaluated cell viability using the MTS assay. The range of concentrations of the Cy3_HyP_LNA/2’OMe probe tested on AGS cells, between 0.4 μM and 2 μM did not affect cell viability, since no statistically significant differences were found for any of the concentrations tested (p > 0.05) relatively to the untreated cells (Fig 2).

### Evaluation of genotoxicity of the HyP_LNA/2’OMe probe by VITOTOX®Assay

**Fig 3:**
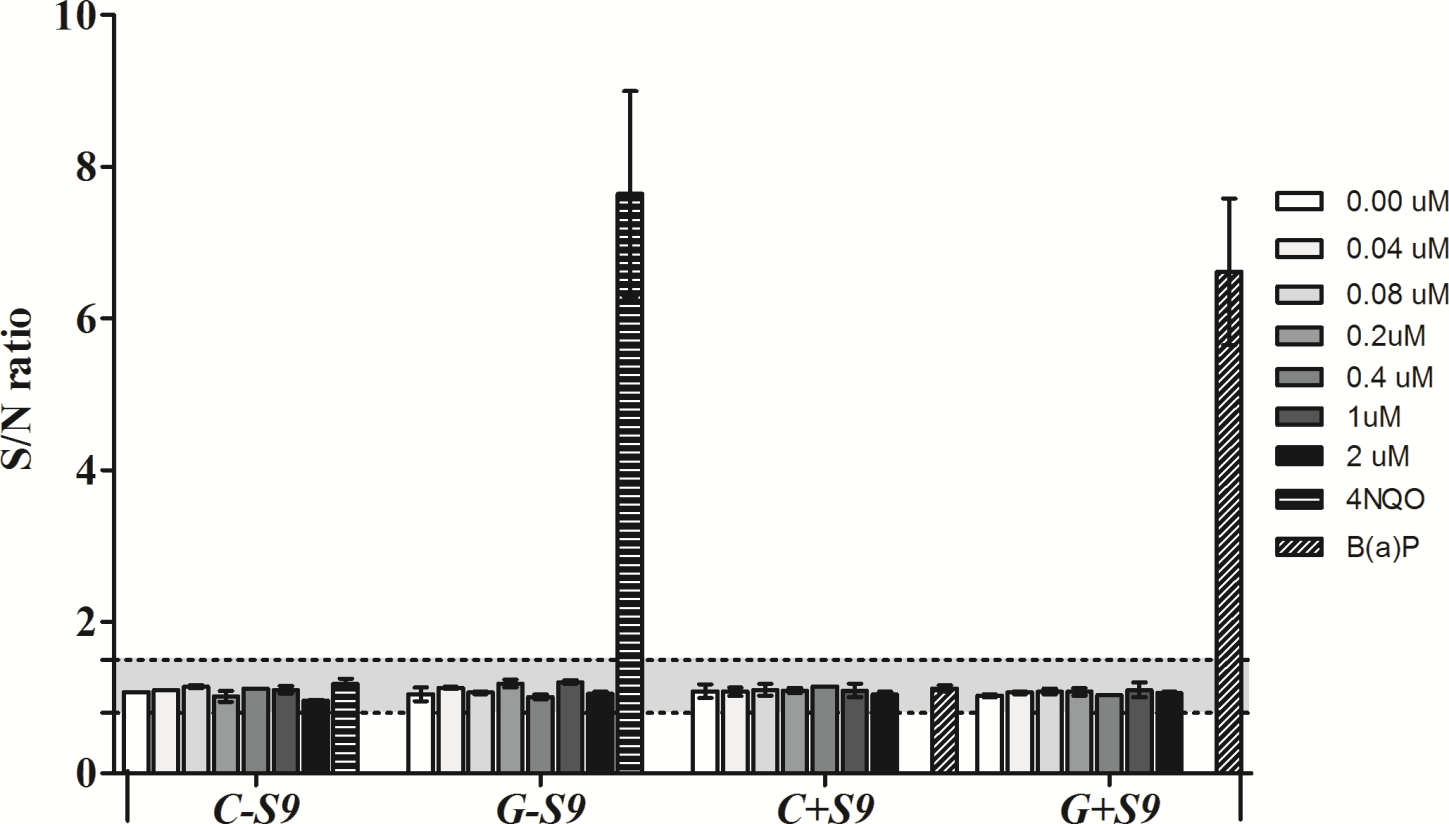
VITOTOX^®^ assay for detection of signs of genotoxicity caused by the Cy3_HyP_LNA/2’OMe probe. Results are expressed as the signal-to-noise (S/N) *ratio* between exposed and unexposed VITOTOX^®^ test bacteria (Genox or Cytox strain) in the absence or presence of S9 mix. Bap:benzo[*α*]pyrene, the positive control, only turns genotoxic after S9 metabolisation.

For detecting a possible DNA damage (genotoxicicty) caused by the HyP_LNA/2’OMe probe, the VITOTOX® assay was used. This assay allows to study the presence of genotoxicity by measuring the activation of the SOS DNA-repair system induced by genotoxic compounds [24,25]. A Genox/Cytox ratio higher than 1.5 indicates the presence of a genotoxic compound. Two positive controls 4-NQO (positive control without S9 activation) and B(α)P (positive control with S9 activation) were used in this assay [25,26]. The results from the VITOTOX® assay are shown in Fig 3. A serial dilution of the probe was tested ranging from 0.04 μM to 2 μM. The tested concentrations of the probe did not reduce the signal to noise ratio of the treated cytox cultures below 0.8 (C in Fig 3). This was also the case after addition of the S9 liver extract in the TA104 pr1 strain, indicating lack of toxicity towards the *S*. *typhymurium* model and enabling to test for genotoxicity. The signal to noise ratio of the luminescence emitted by the genox strain did not exceeded 1.5 before and after addition of the S9 liver extract (G in Fig 3). At the tested concentrations, the activation of the SOS DNA repair mechanism and early signs of genotoxicity elicited by the Cy3_HyP_LNA/2’OMe probe or its possible metabolites could not be observed.

### Fluorescence in situ hybridization (FISH) of Cy3_HP_LNA/2OMe_PS probe on *H*. *pylori* SS1 smears

**Fig 4:**
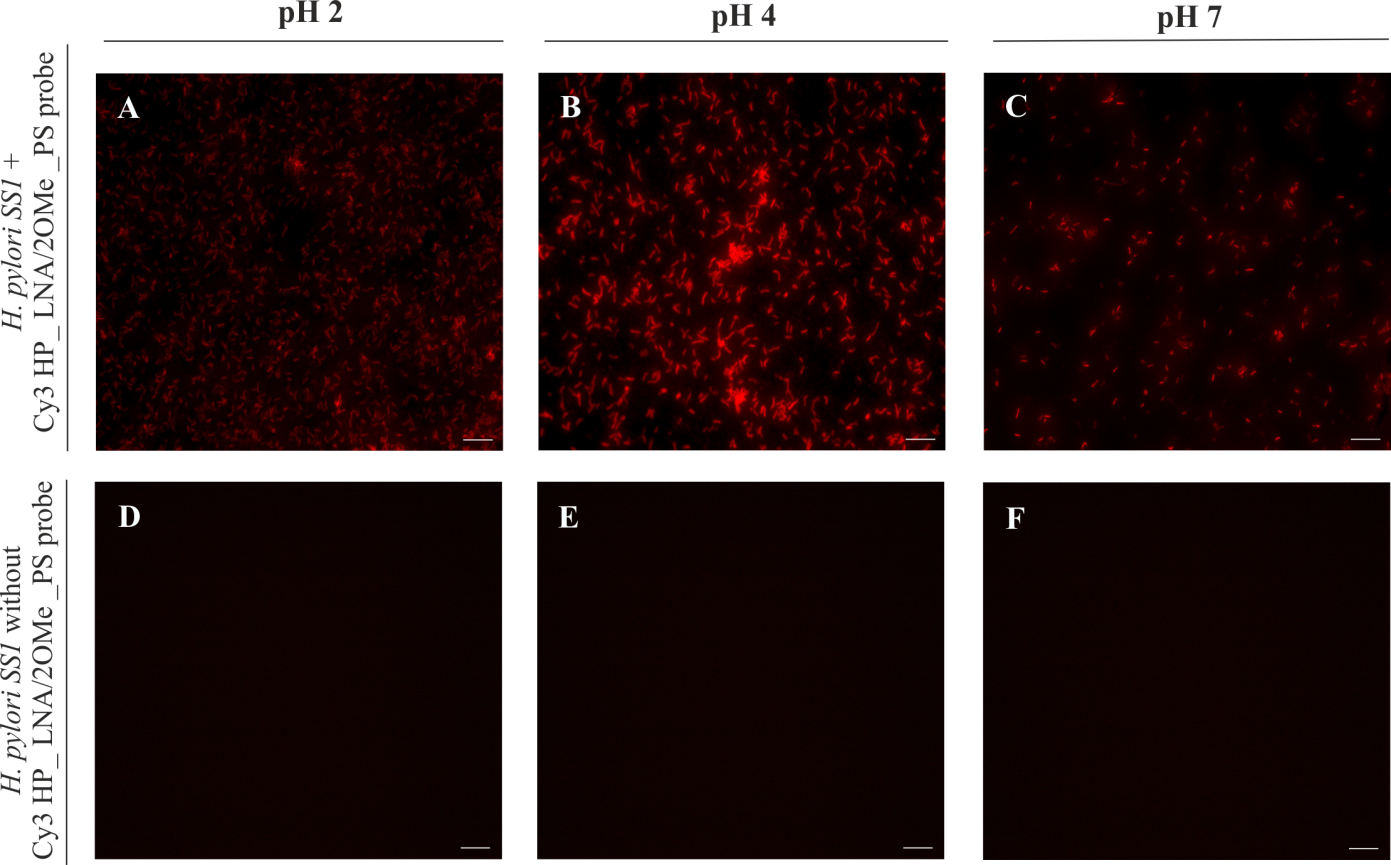
Detection of *H*. *pylori SS1* in slides, by fluorescence *in situ* hybridization (FISH) using the Cy3_HP_LNA/2OMe_PS probe at different pH values, analyzed by epifluorescence microscopy. A to C—FISH protocol performed on smears of pure cultures of *H*. *pylori* SS1 incubated with 0.2 μM of probe. D to F—FISH protocol on smears of *H*. *pylori* SS1 cultures without probe used as negative control. A and D—Experiments performed at pH 2. B and E—Experiments performed at pH 4. C and F—Experiments performed at pH 7. All images were taken at equal exposure times. Scale bar: 10 μm.

To evaluate whether the Cy3_HP_LNA/2OMe_PS probe is able to detect *H*. *pylori* SS1 we performed FISH in smears, at different pH values of the hybridization solutions. As it can be observed in Fig 4, a high fluorescent signal was detected in *H*. *pylori* SS1 smears at all conditions tested. This is similar to what we have reported before for smears of other strains of *H*. *pylori* [22] with, in which high fluorescence was obtained at pH 4 using a non-fixative protocol.

### Bacteria colonization of the gastric mucosa in C57BL/6 mice

**Fig 5:**
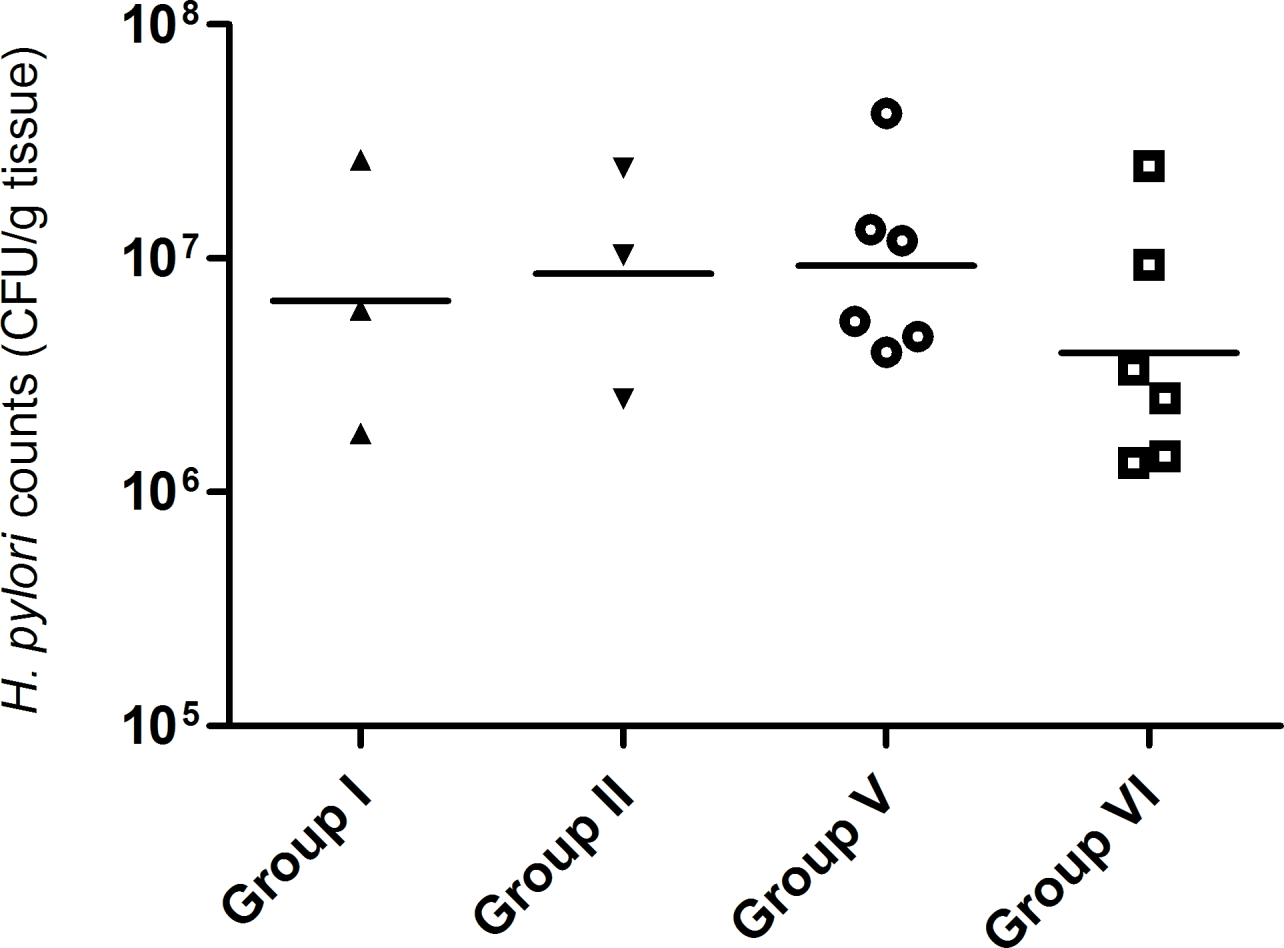
Viable bacterial counts (CFU, colony-forming units) from stomachs of mice infected with *H*. *pylori* SS1 strain for 2 weeks. Scatter plot of CFU for each animal, with bars representing the mean value.

Before the detection of *H*. *pylori* SS1-infecting C57BL/6 mice by FIVH, we assessed the efficiency of colonization in the experimental animal groups to assure that any difference in FIVH signal was not attributed to differences in the colonization levels of bacteria. For that, we quantified the bacterial burden in the stomach of each infected mice by counting the CFU per gram of stomach tissue (Fig 5). At 2 weeks of infection, all mice had an established infection. No differences in bacterial burden were found in the stomachs of infected animals, since CFU levels were equivalent regardless of delivery or not of probe and vehicle. Further, no *H*. *pylori* were found in the non-infected control animals.

### Assessment of *H*. *pylori* in the mice stomach by FIVH

**Fig 6:**
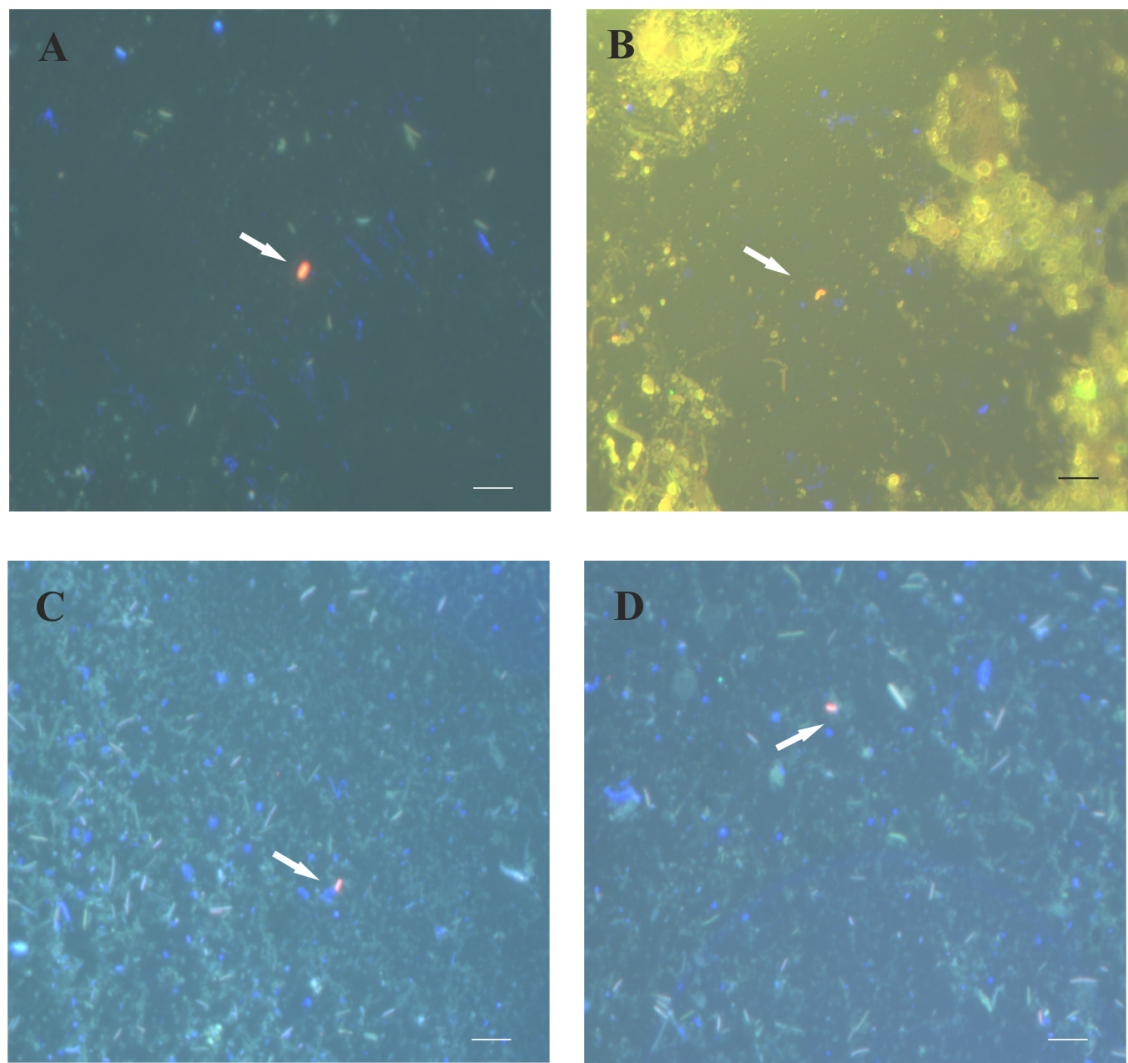
Detection of *H*. *pylori SS1* in the surface of gastric mucus from mice subjected to FIVH with 0.5 μM (A and B; group V) and 2 μM (C and D; group VI) of the Cy3_HP_LNA/2OMe_PS probe. Samples were collected and visualized directly using the epifluorescence microscope. Arrows indicate free-swimming *H*. *pylori* in the surface of mice gastric mucus. All images were taken at equal exposure times and are representative of the respective test group. Channels red, green and DAPI are overlapped. Scale bars: 10 μm.

**Fig 7:**
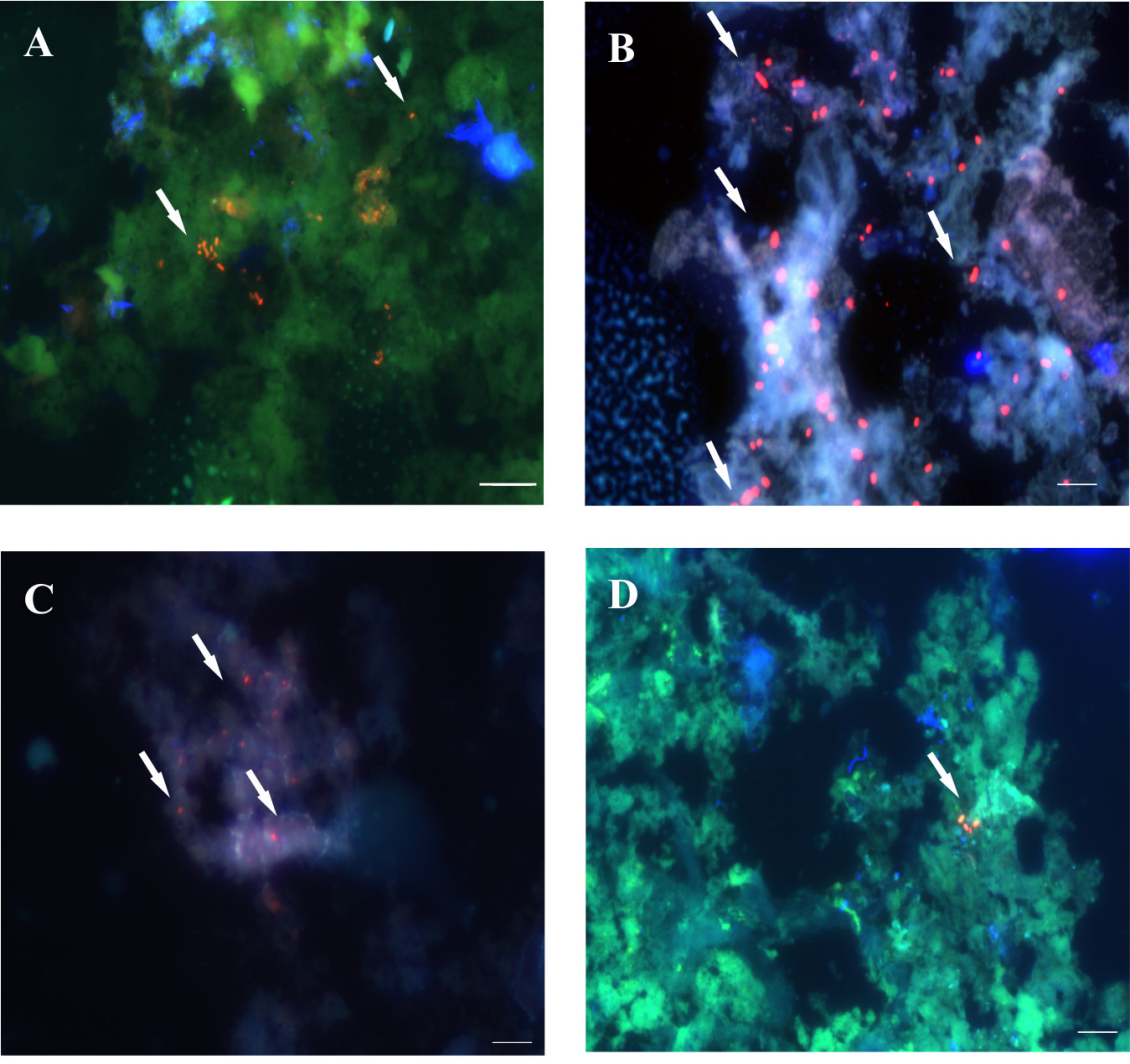
Detection of *H*. *pylori SS1* in frozen sections of gastric mucosa from mice subjected to FIVH, 30 min before euthanasia. A and B: mice from group V (0.5 μM of the Cy3_HP_LNA/2OMe_PS probe), C and D: group VI (2 μM of the Cy3_HP_LNA/2OMe _PS probe). Arrows indicate the presence of *H*. *pylori* infecting the gastric mucosa. All images were taken at equal exposure times, with overlapping of the red, green and DAPI channels. All the images from each group are representative of *n* = 6 mice. A and C- scale bars: 50 μm. B and D- scale bars: 10 μm.

**Fig 8:**
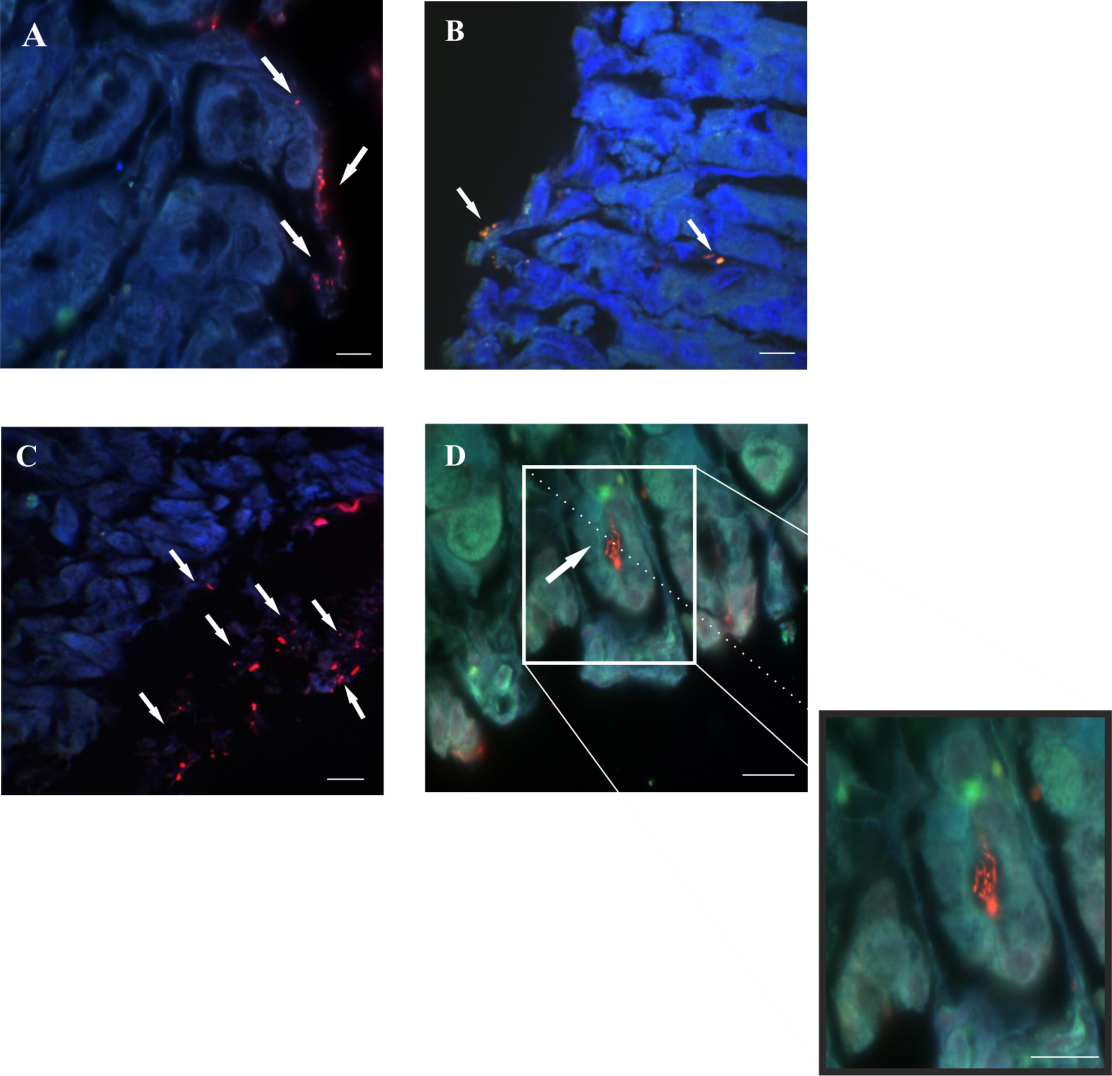
Detection of *H*. *pylori SS1* in paraffin sections of gastric mucosa from mice test groups subjected to FIVH with the Cy3_HP_LNA/2OMe_PS probe 30 min before being sacrificed. A and C. Fluorescence images of the detection in the surface of the gastric mucosa. B and D. Fluorescence images of the detection in the epithelium. A-B images obtained from mouse stomachs from group V. C-D images obtained from mouse stomachs from group VI. Red, green and DAPI channels are overlapped. All the images from each group are representative of *n* = 6 mice. A, B and C scale bars: 10 μm; D scale bars: 50 μm.

Having demonstrated that *H*. *pylori* SS1 colonization of C57BL/6 mice was similar in all animal groups tested (Fig 5), we assessed the efficiency of FIVH experiments by microscopical observation of both mucus samples (surface mucus layer) and mucosa sections from each mouse. At 2-weeks post-infection, it was possible to observe free-swimming *H*. *pylori* within the surface of the mice mucus (Fig 6), as well as attached to gastric epithelial cells (Figs 7 and 8), the major locations of *H*. *pylori* infection in the stomach [28], as identified by the fluorescent signal of the probe. Both concentrations of the Cy3_HP_LNA/2OMe_PS probe studied 0.5 μM (Fig 6A and 6B) and 2 μM (Fig 6D and 6E) were effective in FIVH for the mucus samples of test groups V and VI. Surface mucus samples collected from infected control groups without probe (groups I and II) and from uninfected control groups (groups III and IV) showed no detectable fluorescence emission in the red channel (S1 Fig).

Next, for the detection of *H*. *pylori* SS1 in the gastric mucosa of mice, we used both cryosections and paraffin-embedded sections of the mice stomach. It has been described that, although physically less stable, cryosections are generally superior for the preservation of the fluorescence signals and therefore for detection by microscopy [29]. As it can be observed in Figs 7 and 8, the detection of *H*. *pylori SS1* infection, using FIVH with the Cy3-labeled HP_LNA/2OMe_PS probe, was successful in both frozen and paraffin-embedded stomach sections of the infected test groups V (0.5 μM probe) and VI (2 μM probe).

Although the Cy3_HP_LNA/2OMe _PS probe was effective at detecting bacteria at both concentrations tested, the bacterial morphology was better defined when higher concentration of the probe was used in the case of paraffin-embedded sections (Fig 8C and 8D). In paraffin-embedded sections it was possible to detect free-swimming bacteria in the mucus layer nearby the surface of epithelial cells, as well as *H*. *pylori* adhered to the surface mucus cells (Fig 8A–8C). Furthermore, it was possible to visualize *H*. *pylori* colonizing the glands (Fig 8B and 8D).

A low background in the red channel was observed in infected controls groups (group I and II) in cryosections (S2A–S2D Fig), whereas no detectable red fluorescence emission was found in paraffin-embedded sections (S3A and S3B Fig). No red fluorescent signal (non-specific signal) was observed in non-infected control groups where the Cy3-labeled probe was administrated without *H*. *pylori* (S2E–S2H Fig for cryosections, and S3C and S3D Fig for paraffin-embedded section; group III and IV).

**Fig 9:**
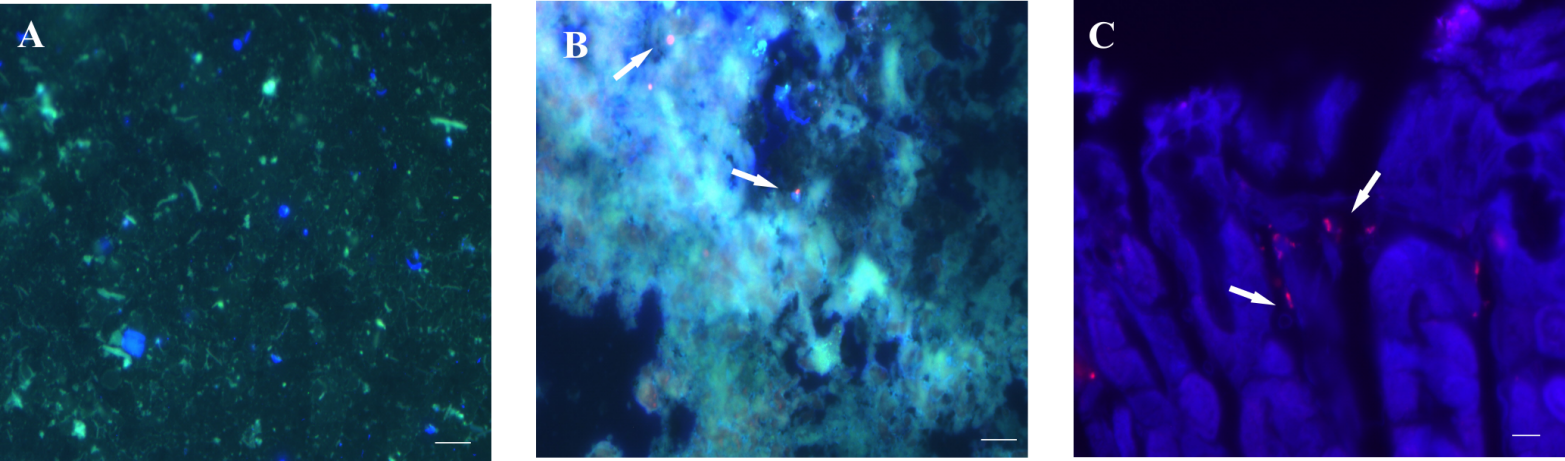
Retention of the Cy3_HP_LNA/2OMe_PS probe in the mouse stomach infected with *H*. *pylori* SS1 for 5 days, and subjected to a FIVH period of 24 hrs before being sacrificed. A. Sample of gastric mucus. B. Frozen section of mouse gastric mucosa. C. Paraffin section of mouse gastric mucosa. Detection of *H*. *pylori SS1* by the Cy3-labeled probe is depicted by white arrows. A and B: Red, green and DAPI channels were overlapped. C. Red and DAPI channels are overlapped. All the images from each group are representative of *n* = 3 mice. Scale bars: 10 μm.

To analyze the retention time of the Cy3_HP_LNA/2OMe_PS probe in the gastric mucosa after FIVH, we increased the period post-FIVH from 30 min to 24 h. The probe was administrated at a concentration of 2 μM to a group of *3* mice at day 5 post-infection. After 24 h of the FIVH, mice were sacrificed and the infection was confirmed by counting the bacterial CFUs (S4 Fig), and the stomach of each mouse was analyzed using fluorescence signal (Fig 9). The number of CFUs obtained from the stomach of mice with a 5 days-infection (S4 Fig) was lower than that obtained from mice with a 15 days-infection. No signal was observed in the red channel in the surface of the mice mucus (Fig 9A), and few bacteria were observed in frozen sections (Fig 9B). In contrast, in paraffin-embedded sections it was possible to detect *H*. *pylori* adhered to gastric epithelial cells (Fig 9C), indicating that the probe was retained in the stomach, though the fluorescence intensity was rather lower (Fig 9B and 9C).

## Discussion

In the present study, we evaluated the efficiency of detection of *H*. *pylori* using *in vivo* labeling with a Cy3_HP_LNA/2OMe_PS probe directed against the 16S rRNA sequence, by FIVH on a mouse model. C57BL/6 mice were infected for 15 days with *H*. *pylori* SS1 strain and FIVH was performed 30 min before mice were sacrificed. Our results demonstrated that *H*. *pylori* can be successfully detected in the gastric mucosa of mice. Moreover, the Cy3_HP_LNA/2OMe_PS probe showed high *in vivo* specificity with low background.

The detection of molecules in living cells constitutes an important field of study in biology and medical diagnostics. Therefore, efforts have been carried out to develop specific methods to target different molecules, such as proteins, mRNA, miRNA, and rRNA, mainly based on fluorescent probes [30,31,32,33]. Successful detection of fluorescent probes depend on several characteristics, namely: high fluorescence level and specificity, high *in vivo* stability and high spatial resolution [34]. The FIVH term was introduced by Wiegant *et al*. in 2010, to described a method to microinject a 2’OMe probe into live eukaryotic cells in culture [35]. Later on, other studies have also used fluorescence labeled oligonucleotides to target or regulate molecules *in vivo* [32,36,37,38]. This *in vivo* labeling methodology can be applied to address the challenge of a fast diagnosis of microbial infections in humans. The aim is to use FIVH in the future, for real-time detection of *H*. *pylori* during upper endoscopy using confocal laser endomicroscopy (CLE) [5].

LNA probes have been applied *in vivo* mainly for therapeutic purposes [39,40]. To the best of our knowledge, there are no *in vivo* studies that use LNA probes for diagnostic purposes.

Previously, we reported that the Cy3_HP_LNA/2OMe_PS probe was non-toxic at the concentration of 0.2 μM against the gastric AGS cell line [22]. In the present study, higher concentrations of the probe were needed for *in vivo* experiments. Therefore, viability analysis using a range of concentration between 0.4 μM and 2 μM of the Cy3_ HP_ LNA/2OMe _PS probe was performed in AGS cells. Although selected studies have demonstrated some toxicity associated to LNA probes [41,42], no statistically significant changes in cell viability were observed within the range of probe concentrations tested in this study. Further, the possibility of genotoxic effects induced by the Cy3_HP_LNA/2OMe_PS probe was also investigated. According to the results, the probe was not genotoxic, even at high concentrations. To our knowledge this is the first report that tests the genotoxicity of LNA probes. Since the VITOTOX^®^ test is known to correlate very well with the Ames assay, which is essentially a gene mutation assay [43], we can conclude that the Cy3_HP_LNA/2OMe_PS probe does not induce genotoxicity by aSOS-induction mechanism.

Additionally, the detection of *H*. *pylori SS1* strain by the Cy3_HP_LNA/2OMe_PS probe was confirmed by FISH on smears of pure cultures of this bacterium, using a range of pH conditions, as well as with a simplified protocol without permeabilization. Although this step is crucial for FISH in bacterial suspensions as we previously reported [22], it is dispensable when using bacteria smears. This simplification of the protocol is relevant for *in vivo* experiments, since it will reduce animal manipulations, time and toxicity of the reagents used.

Regarding the *in vivo* detection of *H*. *pylori*, our results have shown that the Cy3_HP_ LNA/2OMe_PS probe was effective in detecting *H*. *pylori*-infected mice at both probe concentrations tested (0.5 μM and 2 μM), and in different locations (within the mucus and attached to gastric epithelial cells). Although we were able to detect *H*. *pylori* in the surface of the mucus samples collected from infected mice, we did not observe a high concentration of bacteria at this location (Fig 6). Contrastingly, a high density of *H*. *pylori* was found in the mucus layer nearby the stomach surface (e.g. Fig 8A and Fig 8C) or attached to the surface of epithelial cells (e.g. Fig 8B). This is in agreement with already published reports [28,44,45,46]. For instance, Schreiber *et al*., described that *H*. *pylori* colonizes the mucus layer located 0–25 μm above the tissue surface and that the remaining part of the mucus layer was almost free of bacteria [28]. In fact, one cannot expect a substantial amount of *H*. *pylori* cells to be present in the surface of the mucus, where the pH is low and proximate to the lumen pH, thus an adverse niche for the maintenance and replication of *H*. *pylori*.

We can also conclude that the Cy3_HP_LNA/2OMe_PS probe, administered by oral gavage, had a good retention 24 hrs post-FIVH (Fig 9C) in the mice stomach lining and was able to diffuse into deeper regions of the mice gastric epithelium (Fig 8), since it detected *H*. *pylori* in the inner mucus layer and at the entrance of gastric pits. It is accepted that the presence of *H*. *pylori* closer to the epithelial surface probably facilitates exchanges between the adherent and swimming populations of bacteria [28,47].

Importantly, and as mentioned above, the Cy3_HP_LNA/2OMe_PS probe is able to detect microorganisms in the close vicinity of the gastric epithelium, without being affected by extreme conditions of the stomach, as we suggested in our previous study [22]. Thus, the mice stomach environment was not an obstacle for the detection of *H*. *pylori* using our probe, administered by oral gavage, and the FIVH conditions tested here. However, one of the variables that might affect the detection by FIVH is the amount of *H*. *pylori* that are infecting the stomach. In this study, we have achieved standard levels of contamination by this bacterium (Fig 5). The adaptability of this method for situations where a low level of infection exists was not studied.

Improvements in the methodology still have to be addressed in the future in order to include FIVH in the routine clinical practice. The use of CLE is at the moment limited to the equipment available in the market that have an excitation wavelength of 488 nm, which is not compatible with the Cy3 fluorochrome (wavelength around 530–560 nm). Thus, it will be necessary to modify the fluorochrome of the probe. In our previous study [22], we showed that fluorescein-labeled probes had low fluorescence when exposed to gastric fluid juice. Therefore, our future work will comprise selection of a different label not only compatible with CLE devices, but also that preserves characteristics such as high brightness, low photobleaching, higher biostability, low toxicity, and a competitive price. Overall, and although there are still some limitations that need to be addressed, the FIVH methodology described herein represents a remarkable advance towards the *in vivo* diagnosis of *H*. *pylori* infection. Adding to the possibility of detecting pathogens *in vivo*, FIVH will also allow to localize microorganisms directly within the human body. At the moment, a large number of studies have associated gut microbiota with different diseases [48,49], but there are no available technologies to visualize directly the microorganisms and asses their spatial interactions with other microorganisms and with human tissues. The application of FIVH would hence allow to complement the knowledge on the population structure with information about preferred locations of the microorganisms, with a final goal of better understanding the physical-chemical interactions between all the cells of the human complex ecosystem.

In summary, we have evaluated the *in vivo* diagnostic potential of a LNA based probe for the detection of *H*. *pylori* by FIVH in an infected C57BL/6 mouse model. Overall, our results showed that the Cy3-labeled HP_LNA/2OMe_PS probe using the developed FIVH protocol allows the detection *H*. *pylori*, both within gastric mucus and attached to gastric epithelial cells. This holds great promise towards an effective, safe, and immediate method for *in vivo* diagnosis of *H*. *pylori* infection in human patients, applying a confocal laser endomicroscope. Moreover, this methodology can be applied to the study of the detection patterns of bacteria to different anatomic sites that may be relevant during infection processes and for the identification and localization of different components of the microbiome.

## Supporting Information

S1 Fig Analysis of the specificity of Cy3 HP_ LNA/2OMe _PS probe and the background *in vivo* in samples of surface gastric mucus from mice control groups. Samples were collected and analyzed directly under the epifluorescence microscope. A and B: group I, C and D: group II, E and F: group III, G and H: group IV. All images were taken at equal exposure times. Channel red, green and DAPI were overlapping. Scale bars: 10 μm.
(TIFF)

S2 Fig Analysis of the specificity of Cy3 HP_ LNA/2OMe _PS probe and the background *in vivo* in cryosections from gastric mucosa of control groups. A and B: group I, C and D: group II, E and F: group III, G and H: group IV. All the images are representative of *n* = 3 mice. All images were taken at equal exposure times. Channel red, green and DAPI were overlapping. Scale bars: 10 μm.
(TIF)

S3 Fig Analysis of specificity of Cy3 HP_ LNA/2OMe and background in paraffin sections of mouse stomach from control groups. A-B: Background obtained using the vehicle and water from group I and II, respectively. C—D. Distribution of Cy3 HP_ LNA/2OMe _PS probe in mouse stomach of group III and IV, respectively. All the images are representative of *n* = 3 mice. All images were taken at equal exposure times. Channel red, green and DAPI were overlapping. Scale bars: 50 μm.
(TIF)

S4 Fig Quantification of bacterial burden in the stomach of *H*. *pylori*-infected mice with 5 days of post-infection. Bars represent medium values.
(TIF)
