# Supplementary figures and images for: Fluorescence In Vivo Hybridization (FIVH) for Detection of Helicobacter pylori Infection in a C57BL/6 Mouse Model
Source: PLoS One. 2016 Feb 5;11(2):e0148353. doi: 10.1371/journal.pone.0148353 (PMC4743915; doi:10.1371/journal.pone.0148353)

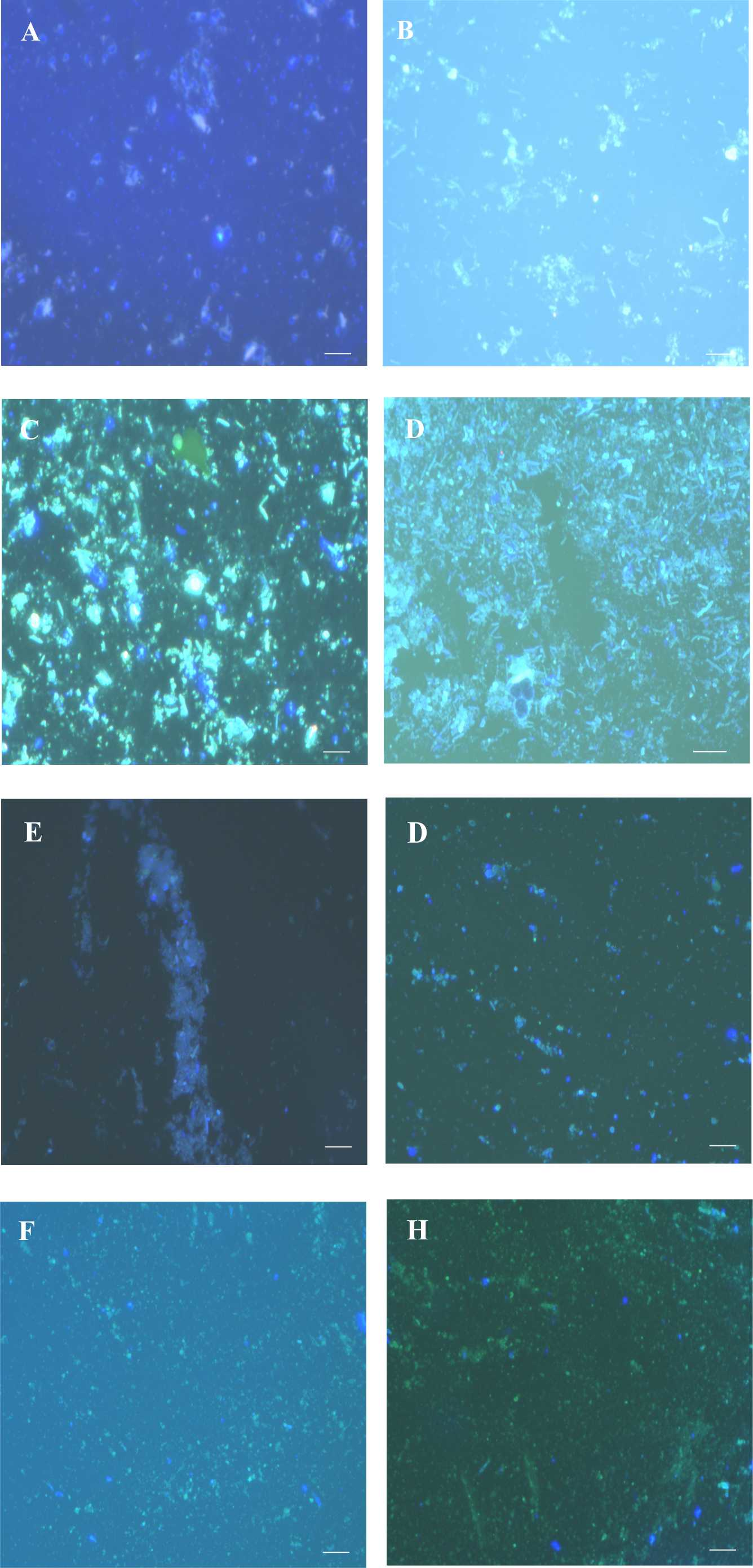

Supplement: S1 Fig — Samples were collected and analyzed directly under the epifluorescence microscope. A and B: group I, C and D: group II, E and F: group III, G and H: group IV. All images were taken at equal exposure times. Channel red, green and DAPI were overlapping. Scale bars: 10 μm. (TIFF) [file pone.0148353.s001.tiff]

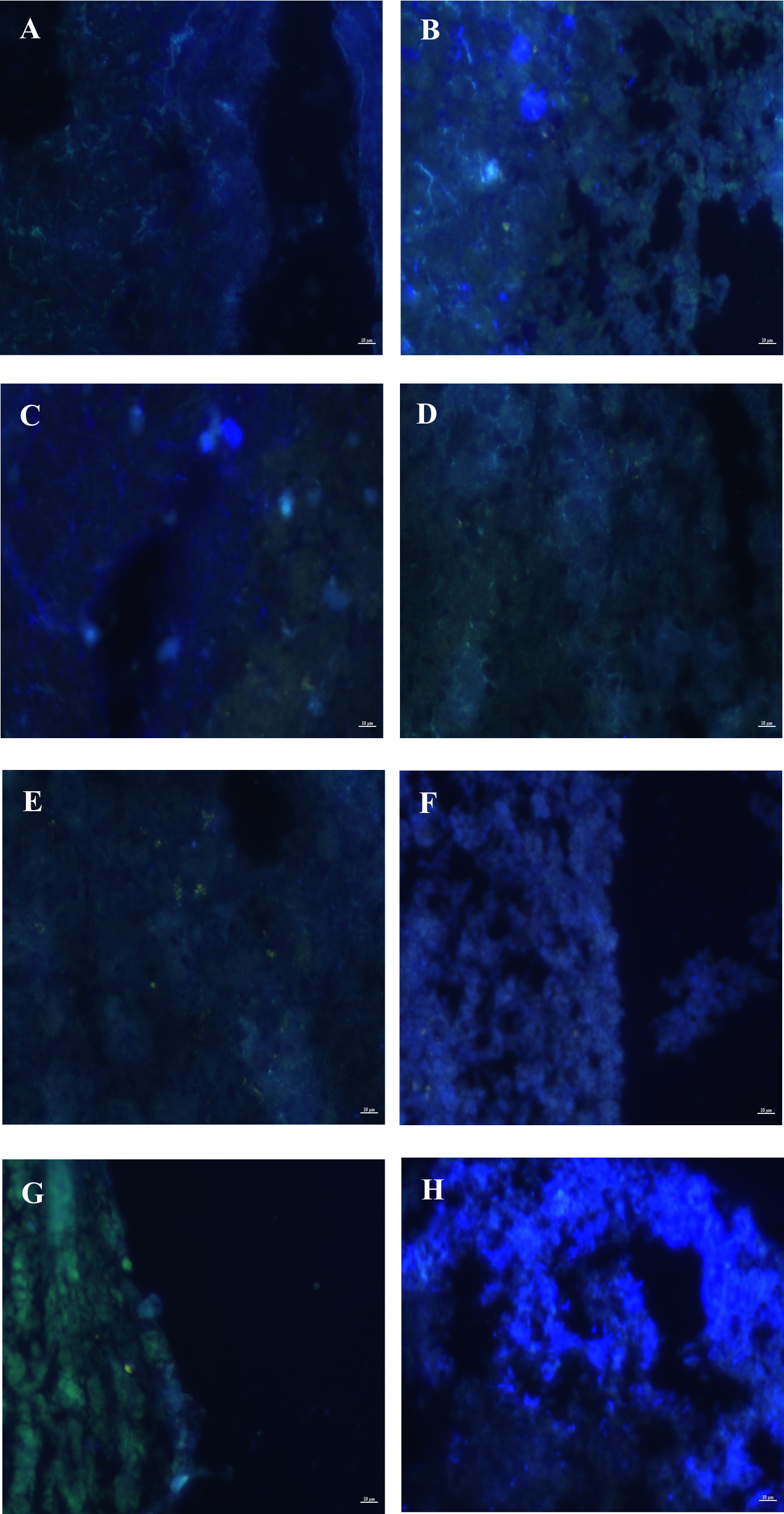

Supplement: S2 Fig — A and B: group I, C and D: group II, E and F: group III, G and H: group IV. All the images are representative of n = 3 mice. All images were taken at equal exposure times. Channel red, green and DAPI were overlapping. Scale bars: 10 μm. (TIF) [file pone.0148353.s002.tif]

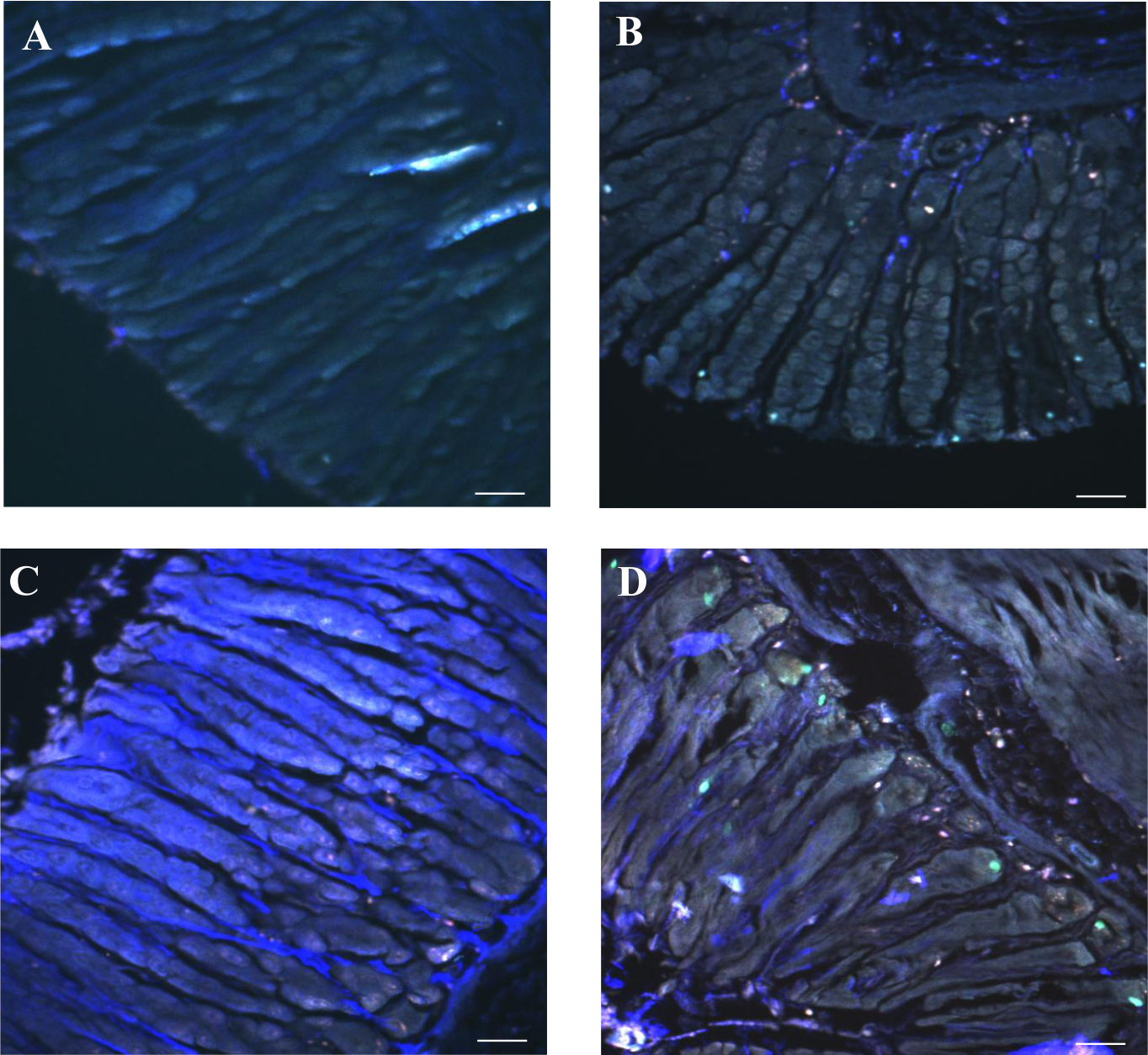

Supplement: S3 Fig — A-B: Background obtained using the vehicle and water from group I and II, respectively. C—D. Distribution of Cy3 HP_ LNA/2OMe _PS probe in mouse stomach of group III and IV, respectively. All the images are representative of n = 3 mice. All images were taken at equal exposure times. Channel red, green and DAPI were overlapping. Scale bars: 50 μm. (TIF) [file pone.0148353.s003.tif]

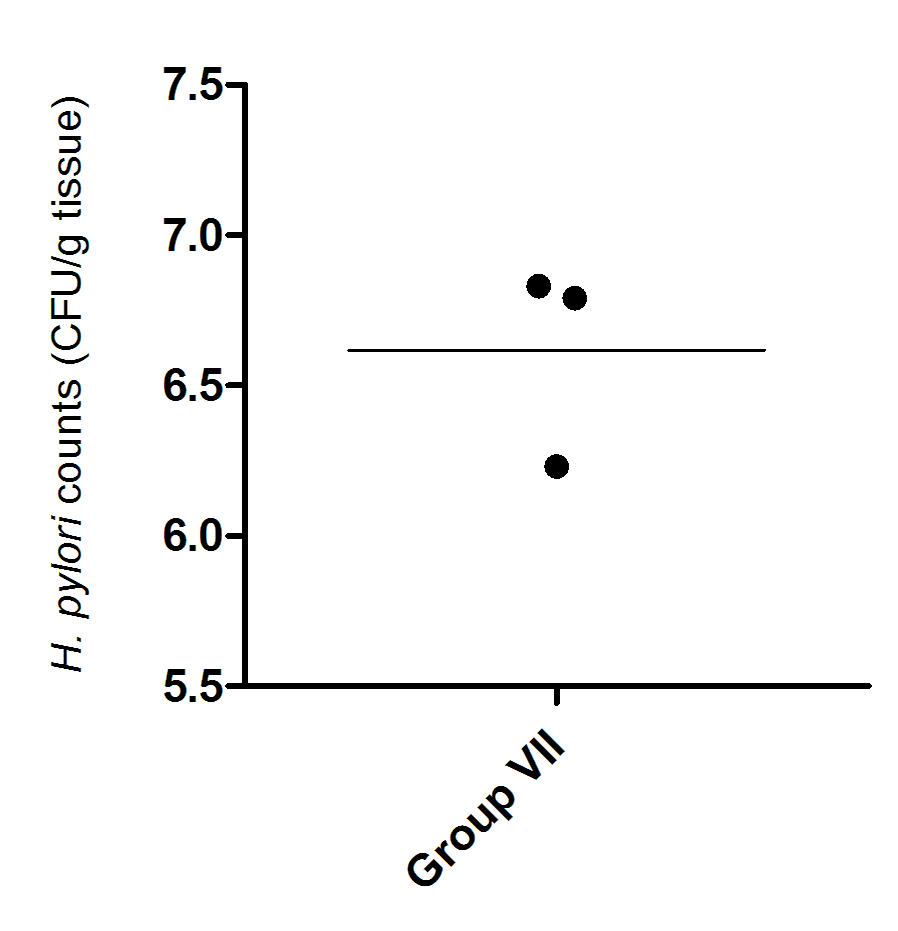

Supplement: S4 Fig — Bars represent medium values. (TIF) [file pone.0148353.s004.tif]
